# Respiratory Failure in Hyperthyroidism: Focus on Thyrotoxic Periodic Paralysis

**DOI:** 10.7759/cureus.90200

**Published:** 2025-08-16

**Authors:** Tun Tun, Wyut Yi Htay, Kaung Myat

**Affiliations:** 1 Horton Hospital, Oxford University Hospitals NHS Foundation Trust, Banbury, GBR; 2 Care of the Elderly, West Middlesex University Hospital, London, GBR; 3 Cardiology, Queen Elizabeth Hospital, London, GBR

**Keywords:** acute respiratory failure (arf), hyperthyroidism, hypokalaemia, thyroid-storm, thyrotoxic periodic paralysis

## Abstract

Thyrotoxic periodic paralysis (TPP) is a rare but serious and potentially life-threatening complication of hyperthyroidism. It is typically characterized by acute flaccid muscle weakness of the lower limbs and hypokalemia. While most cases resolve with cautious potassium replacement and appropriate management of thyrotoxicosis, some progress to respiratory failure, significantly worsening the prognosis. This review examines 14 reported cases of TPP complicated by respiratory failure (published between 2004 and 2025), highlighting clinical gaps in early recognition and the atypical presentations that contributed to delayed diagnosis. Additionally, the mechanisms of respiratory compromise in thyroid storm - another thyrotoxic emergency - are discussed to provide a broader understanding of respiratory failure within the spectrum of thyrotoxic states. By analyzing these case reports, this review aims to raise awareness among clinicians of unusual presentations, emphasize the need for early recognition, and advocate for timely multidisciplinary involvement, particularly from respiratory, endocrinology, and intensive care teams, in managing such critical cases.

## Introduction and background

Thyrotoxic periodic paralysis (TPP) is a rare but serious complication of hyperthyroidism, most commonly associated with Graves’ disease. It is characterized by acute, symmetrical proximal lower limb weakness caused by hypokalemia and may progress to involve all four limbs. In rare instances, the respiratory muscles may also be affected. The underlying pathophysiology is thought to involve increased activity of the Na⁺/K⁺-ATPase pump, stimulated by excess thyroid hormones, leading to intracellular potassium shifts and resulting hypokalemia. Although TPP has traditionally been reported more frequently among East Asian males, its incidence is increasingly being recognized in Western and other populations [1].

Failure to promptly recognize and manage TPP may result in life-threatening complications such as respiratory failure and cardiac arrhythmias, including QT prolongation, Torsades de Pointes, and ventricular arrhythmias [2].

While TPP is a distinct clinical entity, respiratory failure can also occur in other hyperthyroid states, such as thyroid storm, a life-threatening exacerbation of thyrotoxicosis involving respiratory muscle fatigue and multisystem dysfunction.

This narrative review focuses on the association between hyperthyroidism and respiratory failure, with particular emphasis on TPP-related respiratory muscle paralysis. The aim is to consolidate the available case-based literature, explore the proposed mechanisms, highlight clinical patterns, and identify existing gaps to promote recognition and management of these under-recognized but potentially fatal manifestations.

## Review

Thyrotoxic periodic paralysis: literature overview

Epidemiology of TPP

TPP has been predominantly reported in Asian populations, with incidence rates of 1.9% among Japanese patients with thyrotoxicosis and 1.8% among Chinese patients [3,4]. It has also been documented in individuals from Thai, Filipino, Vietnamese, Korean, and Malaysian backgrounds [5].

TPP can occur in patients with any form of hyperthyroidism; however, it is most commonly associated with Graves’ disease [2,6,7]. Interestingly, despite the overall higher incidence of thyrotoxicosis in women, TPP occurs predominantly in middle-aged men, with a reported male-to-female ratio of approximately 20:1 [5].

Pathophysiology

The hallmark hypokalemia observed in TPP primarily results from a transcellular shift of potassium into skeletal muscle cells rather than from actual potassium depletion. This intracellular shift is largely driven by increased activity of the Na⁺/K⁺-ATPase pump, which is stimulated by excess thyroid hormones [8]. As skeletal muscle contains the majority of the body’s intracellular potassium, it plays a major role in maintaining extracellular potassium balance. Thyroid hormones have been shown to increase Na⁺/K⁺-ATPase gene expression, facilitating this shift [1,7].

In addition to the effects of thyroid hormones, β-adrenergic stimulation-particularly through β₂-receptors-also contributes to hypokalemia by increasing intracellular cyclic AMP levels, which further enhance Na⁺/K⁺-ATPase activity in muscle cells [9]. Thyroid hormones also upregulate both the density and sensitivity of β-adrenergic receptors, amplifying catecholamine-driven potassium uptake [10].

Moreover, episodes of TPP are frequently associated with hyperinsulinemia, which further stimulates the Na⁺/K⁺-ATPase pump, contributing to the intracellular shift of potassium. This mechanism helps explain why carbohydrate-rich meals are common precipitants of TPP attacks [11].

Hypophosphatemia is another biochemical abnormality often observed during TPP episodes, attributed to a similar transcellular shift of phosphate into muscle cells during attacks [5].

Genetic Insights of TPP

Genetic predisposition plays a significant role in the pathogenesis of TPP. Mutations in genes such as KCNE3, as well as specific human leukocyte antigen (HLA) subtypes (e.g., A2BW22, AW19B17, B5, BW46, DRw8), have been associated with increased Na⁺/K⁺-ATPase activity or altered potassium channel function in skeletal muscle [12].

A major advancement in understanding TPP came with the identification of the KCNJ18 gene, located on chromosome 17p11.1-2. This gene encodes the inward-rectifying potassium channel Kir2.6, which is selectively expressed in skeletal muscle and regulated by thyroid hormone-responsive elements [13]. Mutations in Kir2.6 - found in up to 33% of TPP cases - disrupt normal potassium channel assembly and function, predisposing individuals to intracellular potassium trapping and periodic paralysis [13].

Precipitating Factors

TPP episodes frequently occur during the early morning or late evening hours [5]. One of the most common triggers is a high-carbohydrate meal, which provokes insulin release and promotes potassium uptake into cells [5]. Studies have shown that individuals with TPP exhibit elevated insulin levels both during and between attacks, likely reflecting the hyperadrenergic and hypermetabolic state associated with thyrotoxicosis [14]. Strenuous physical activity is another well-recognized precipitating factor. Following exercise, increased glucose uptake by skeletal muscle, along with compensatory hormonal responses, enhances intracellular potassium shifts and may precipitate an episode of paralysis [14].

Clinical Features

The initial presentation of TPP typically occurs between the ages of 20 and 40 years [5]. It usually begins with symmetrical proximal muscle weakness in the lower limbs and may progress to flaccid quadriplegia. Despite the severity of motor weakness, clinical signs of thyrotoxicosis are often subtle and may be overlooked [5]. Although symptoms frequently resolve spontaneously within a few hours to two days - even without potassium supplementation - serious complications, including cardiac arrhythmias and, in rare cases, respiratory failure, can occur [5].

Evaluation and Diagnosis

Patients with TPP typically demonstrate biochemical evidence of thyrotoxicosis, including elevated levels of free T4 or T3 and suppressed thyroid-stimulating hormone (TSH). Radionuclide thyroid uptake scans often reveal increased uptake, consistent with hyperthyroid states [1]. Hypokalemia is a hallmark feature during paralytic episodes; however, normal potassium levels are not uncommon in some cases, underscoring the diagnostic challenge of TPP [15].

Management of TPP

The treatment of TPP involves two primary goals: acute correction of hypokalemia and long-term control of the underlying thyrotoxicosis. Because total body potassium stores are typically normal, the aim of potassium therapy is to correct serum levels rather than to replenish a true deficit [16].

Immediate potassium supplementation is recommended to accelerate recovery from muscle weakness and to prevent arrhythmic complications. However, close monitoring is essential, as rapid shifts during recovery may lead to rebound hyperkalemia. Clinicians should be aware of the risk of rebound hyperkalemia following potassium supplementation in TPP due to this mechanism. Current evidence suggests that potassium replacement should not exceed 90 mEq within a 24-hour period [17], and some experts advise limiting the infusion rate to no more than 10 mEq per hour to minimize this risk [18]. Therefore, close cardiac monitoring and cautious potassium dosing are essential to prevent potentially life-threatening hyperkalemia

Non-selective beta-blockers, particularly propranolol, are also beneficial due to their ability to counteract β-adrenergic stimulation. Several reports have documented rapid resolution of paralysis following intravenous propranolol, even in cases that were refractory to potassium therapy alone [19-21].

Respiratory failure in thyrotoxic state: a rare but serious complication

TPP is primarily recognized for causing motor weakness in the limbs, involvement of the respiratory muscles can result in acute and potentially life-threatening respiratory failure. This complication most often arises from severe hypokalemia-induced weakness of the diaphragm and accessory respiratory muscles. However, respiratory compromise may also occur in hyperthyroid patients without hypokalemia, typically due to thyrotoxic myopathy, particularly in the context of thyroid storm or longstanding, untreated thyrotoxicosis.

This section explores the distinct mechanisms, clinical presentations, and management strategies associated with respiratory failure in both TPP-related and non-TPP thyrotoxic states. Through a case-based literature review, we aim to highlight key differences as well as shared diagnostic and therapeutic challenges between these two conditions.

Case report review: respiratory failure in TPP

In this narrative review article, the relevant literature and papers were identified by searching PubMed using the keywords 'thyrotoxic periodic paralysis', 'respiratory failure', and 'thyroid storm', 'hypokalemia'. 

As previously discussed, the hallmark presentation of TPP is acute-onset weakness predominantly affecting the lower limbs. However, in rare but potentially life-threatening cases, involvement of the respiratory muscles has also been reported. To examine this atypical manifestation, we reviewed 14 published case reports of TPP-associated respiratory failure between 2004 and 2025. A summary of these cases is presented in Table 1.

**Table 1:** Reviewed case reports of TPP associated with respiratory failure TPP: Thyrotoxic Periodic Paralysis mmol/L: millimole per litre Table created by the authors

No	Study (First Author, Year)	Ethnicity	Age (years)	Sex	Clinical Presentation	Thyroid Pathology	Serum Potassium on Admission (mmol/L)	Ventilatory Support	Outcome
1	Liu et al. (2004) [22]	Chinese	29	Male	Profound weakness of both upper and lower limbs and acute respiratory distress	Hyperthyroidism	1.3	Intubation	Discharge
2	Satam et al. (2007) [23]	Indian	10	Female	Breathing difficulty and myalgia	Hyperthyroidism	4.8	Intubation	Death
3	Wu et al. (2008) [24]	Not reported	29	Male	Generalized convulsion followed by flaccid paralysis of all four limbs after period of prolonged fasting	Hyperthyroidism	1.4	Intubation	Discharge
4	Abbasi et al. (2010) [25]	Korean	27	Male	Psychosis features	Graves’ disease (Newly diagnosed)	2.2 to 2.8	Intubation	Discharge
5	Shields (2015) [26]	African American	43	Female	Recurrent bilateral lower limbs weakness and dyspnoea	Known Graves’ disease	2.8	Intubation	Discharge
6	Naji and Her (2018) [27]	Asian	28	Male	Profound weakness of all four limbs after drinking alcohol	Graves’ disease (Newly diagnosed)	1.6	Intubation	Discharge
7	Qian et al. (2019) [28]	Chinese	29	Male	Flaccid paralysis of all four limbs	Painless Thyroiditis	1.5	Intubation	Discharge
8	Gulati et al. (2020) [29]	African American	25	Male	Bilateral lower extremities weakness	Hyperthyroidism	1.5	Intubation	Discharge
9	Hung et al. (2021) [30]	Not reported	28	Female	Nausea, vomiting and sudden onset respiratory distress and drowsiness	Known hyperthyroidism (Graves’ disease)	1.2	Non invasive Ventilation	Discharge
10	Sethi et al. (2021) [31]	Indian	39	Male	Myalgia, weakness and respiratory distress	Known hyperthyroidism (Graves’ disease)	1.66	Not needed	Discharge
11	Mishra et al. (2023) [32]	Not reported	38	Male	Multiple episodes of vomiting followed by quadriparesis and respiratory distress	Hyperthyroidism (Graves’ disease)	1.7	Intubation	Discharge
12	Son et al. (2023) [33]	Hispanic	31	Male	Acute on chronic back pain and lower extremities paraesthesia then respiratory distress	Hyperthyroidism	1.9	Intubation	Discharge
13	Lalwani et al. (2024) [34]	Not reported	19	Male	Bilateral lower limb weakness, significant agitation	Known hyperthyroidism	1.5	Not reported	Death
14	Song et al. (2025) [35]	Chinese	29	Male	Sudden onset of generalised muscle weakness with significant chest tightness	Graves’ disease (Newly diagnosed)	3.7	Intubation	Discharge

Each case was reviewed with particular attention to patient demographics (including ethnicity, age, and sex), clinical presentation, underlying thyroid pathology, serum potassium levels at admission, relevant comorbidities, drug-related precipitants of respiratory failure, disease timeline and progression, and the requirement for ventilatory support.

Demographics and Patient Characteristics

**Figure 1:**
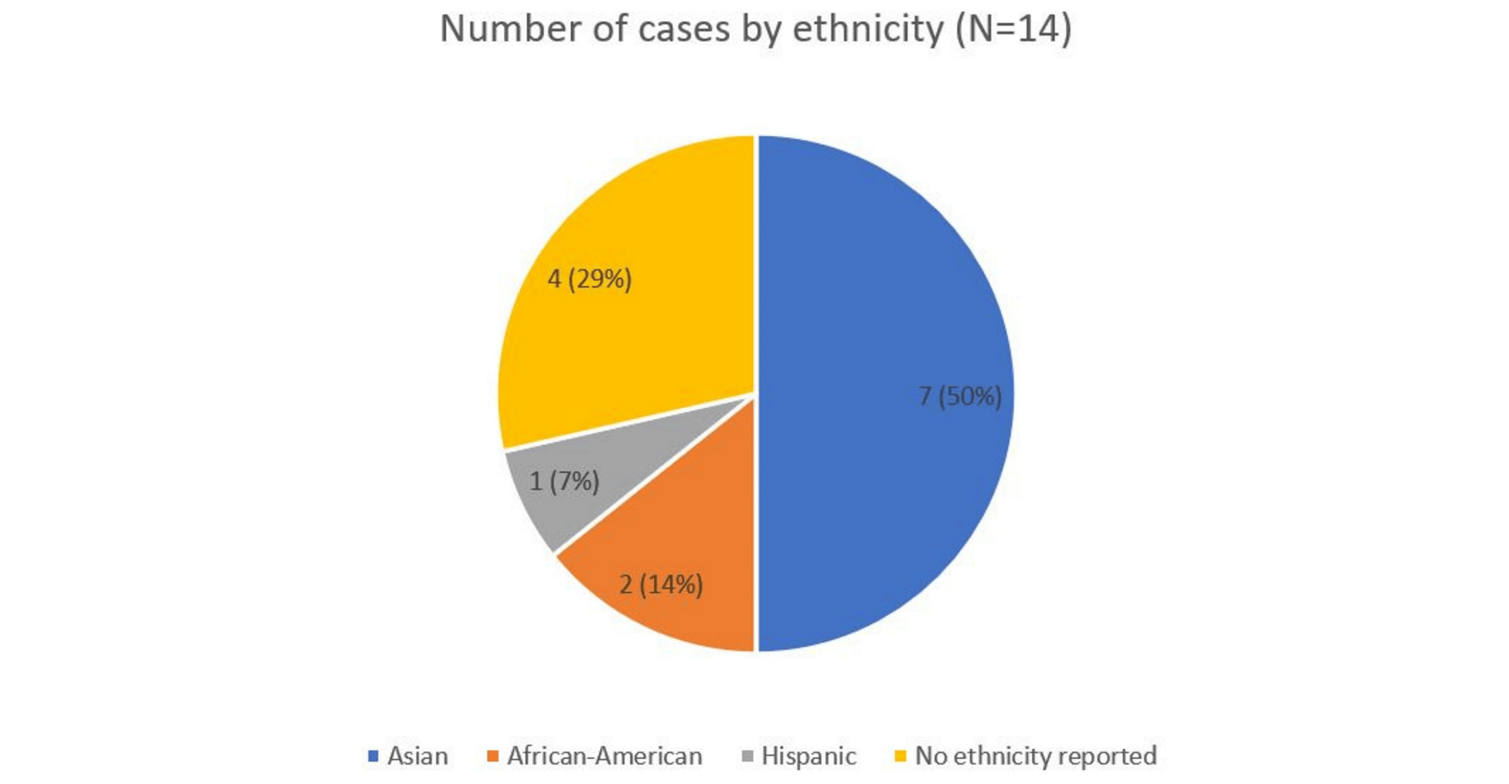
Number of cases by ethnicity (N=14) Figure created by the authors.

A total of 14 reported cases (N = 14) of TPP with respiratory muscle involvement were reviewed. Of these, seven patients (50%) were of Asian origin, two (14%) were African American [26,29], and one (7%) was Hispanic. Ethnicity was not reported in four cases (29%). The ethnic distribution is summarized in Figure 1.

**Figure 2:**
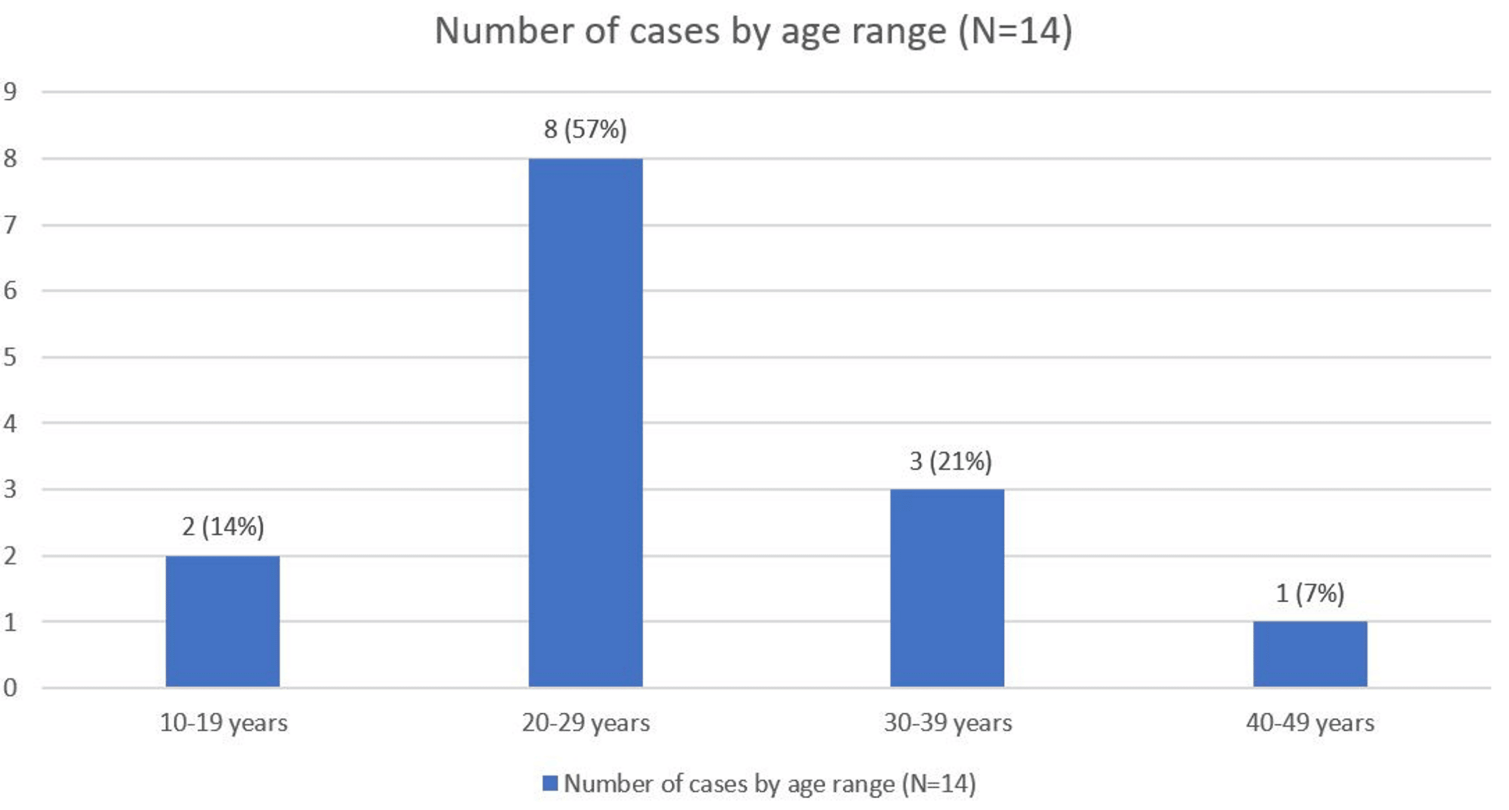
Number of cases by age range (N=14) Figure created by the authors.

The age of patients in the 14 reported cases (N = 14) of TPP with respiratory muscle involvement ranged from 10 to 43 years. The majority of patients (eight, 57%) were between 20 and 29 years old. This was followed by three patients (21%) aged 30-39 years, two patients (14%) aged 10-19 years, and one patient (7%) aged 40-49 years. The age distribution is illustrated in Figure 2.

**Figure 3:**
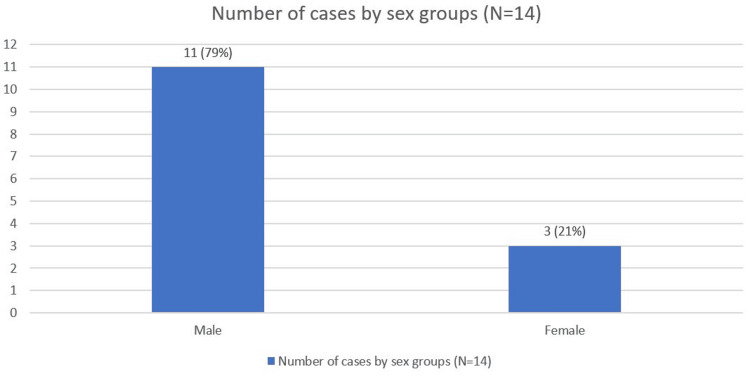
Number of cases by sex group (N=14) Figure created by the authors.

Of the 14 reported cases (N = 14) of TPP with respiratory muscle involvement, 11 patients (79%) were male and three (21%) were female, reflecting that TPP is most commonly seen in male patients. The sex distribution is shown in Figure 3.

Clinical Presentation

While the majority of TPP cases present with limb muscle weakness, several case reports have documented atypical initial symptoms that contributed to delayed diagnosis. In one case, the patient’s initial manifestation was a generalized convulsion, followed by flaccid limb weakness [24]. Another report described a patient who first presented with acute depressive psychotic symptoms, later developing features of thyroid storm, flaccid paralysis, and respiratory muscle involvement [25]. An additional case detailed a patient who initially experienced nausea and vomiting, eventually progressing to respiratory failure [30]. More recently, a patient presented with acute-on-chronic back pain and paresthesia, and subsequently re-presented with respiratory distress [33].

These atypical presentations underscore the clinical variability of TPP and highlight the diagnostic challenges and treatment delays that may occur in the absence of classic signs.

Associated Hyperthyroid Pathology

TPP can occur in the context of various hyperthyroid states, including Graves’ disease. In this review, four out of 14 cases involved patients with a known history of hyperthyroidism [26,30,31,34], while the remaining 10 had no prior diagnosis. Among the 14 cases, six were associated with Graves’ disease [25,27,30-32,35]. In one case reported in 2019, the patient presented with flaccid limb weakness and respiratory failure, and the underlying etiology was identified as painless thyroiditis [28]. The remaining case reports did not fully investigate or specify the underlying cause of thyrotoxicosis.

These findings highlight that TPP with respiratory failure is not exclusive to Graves’ disease and can also occur in other thyrotoxic states, such as thyroiditis. Clinicians should be aware that flaccid muscle weakness - especially when accompanied by respiratory compromise - can be the initial presentation of TPP, even in patients without a known history of thyroid disease. Therefore, thyroid function testing and additional investigations should be considered in any patient presenting with muscle weakness and respiratory failure to determine the underlying etiology.

Serum Potassium Levels on Admission

**Figure 4:**
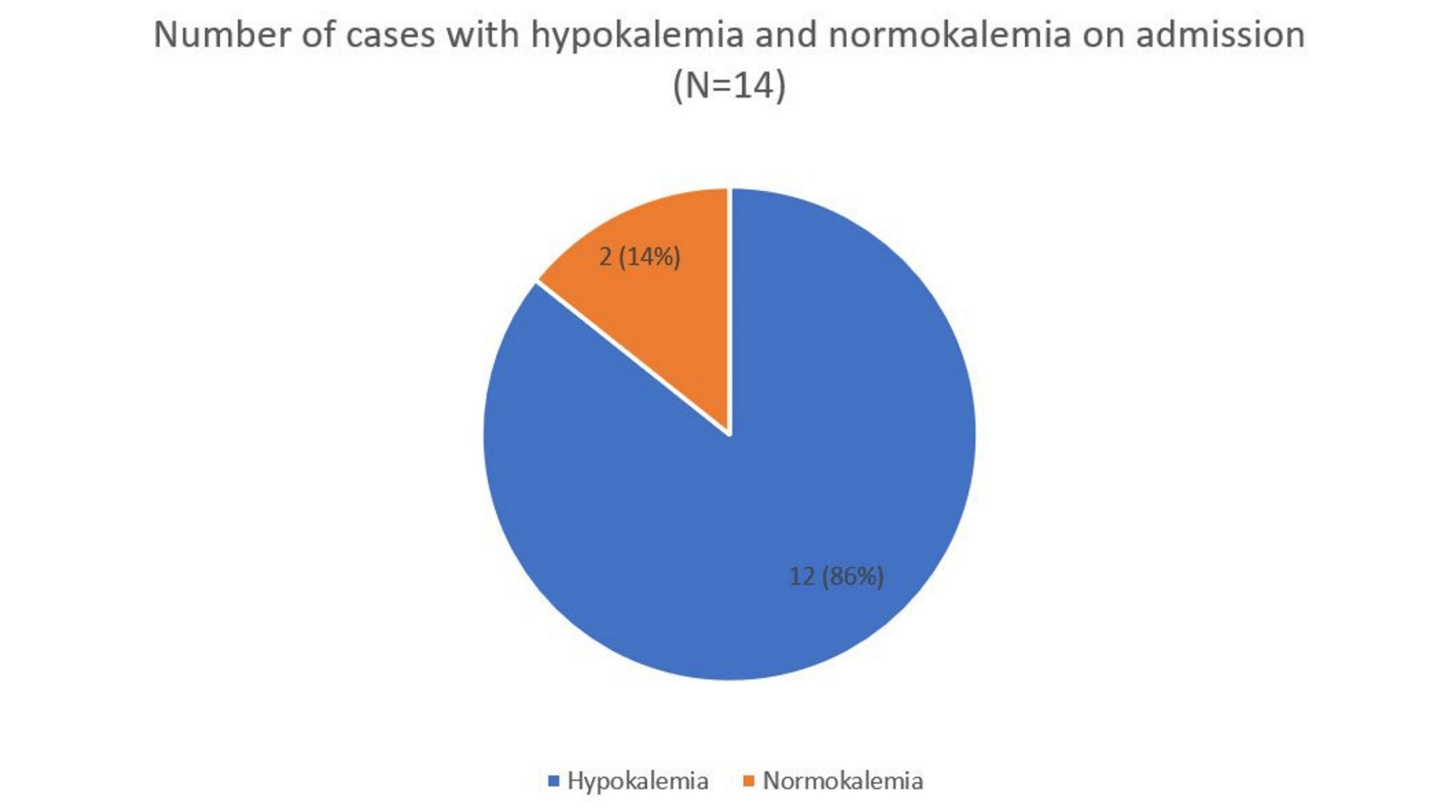
Number of cases with hypokalemia and normokalemia on admission (N=14) Figure created by the authors.

In this review, 12 out of 14 cases of TPP with respiratory muscle involvement were associated with significant hypokalemia, typically with serum potassium levels below 2 mmol/L. However, two case reports described respiratory failure occurring in the presence of normal serum potassium levels above 3.5 mmol/L [23,35]. Figure 4 illustrates the potassium levels of patients on admission. Serum potassium levels on admission were reported in all 14 cases (N = 14). Hypokalemia was present in 12 patients (86%), while two patients (14%) had normal potassium levels. In one case report published in 2007, the patient presented with a normal serum potassium level of 4.8 mmol/L, which contributed to a delay in diagnosis and ultimately resulted in a fatal outcome. Post-mortem biopsy of striated muscle in this case revealed features of thyrotoxic myopathy, which may explain the development of respiratory failure despite normal potassium levels [23].

This case underscores the importance of maintaining a high index of suspicion for TPP in patients presenting with acute limb weakness, even in the absence of hypokalemia. It also supports the recommendation to include thyroid function tests in the initial diagnostic workup for such presentations.

Although the exact mechanism of respiratory muscle involvement in TPP remains incompletely understood, it is believed to be primarily related to hypokalemia-induced muscle dysfunction.

Association With Other Medical Conditions and Drug-Related Respiratory Depression

None of the 14 reviewed cases had a history of underlying cardiac disease, renal impairment, or chronic respiratory conditions such as asthma, chronic obstructive pulmonary disease (COPD), or obstructive sleep apnea. This suggests that respiratory failure in TPP is not typically associated with pre-existing cardiopulmonary or renal comorbidities. Additionally, none of the cases reported respiratory failure precipitated by medication use.

Timing, Progression and Disease Course

A review of the 14 reported cases of TPP with respiratory muscle involvement revealed variability in the timing of respiratory support initiation. Not all case reports specified the exact timing of intubation or extubation; however, based on the available data, the time to intubation ranged from as early as one hour [26] to as late as 10 hours after symptom onset [24]. Extubation was reported to occur as early as 8.5 hours [28] and as late as five days following intubation [35]. This variability reflects a significant clinical gap in the literature and underscores the need for more standardized reporting. Detailed documentation of symptom progression, severity, and the total duration of ventilatory support is essential to better understand and manage respiratory failure in patients with TPP.

Requirement for Respiratory Support

**Figure 5:**
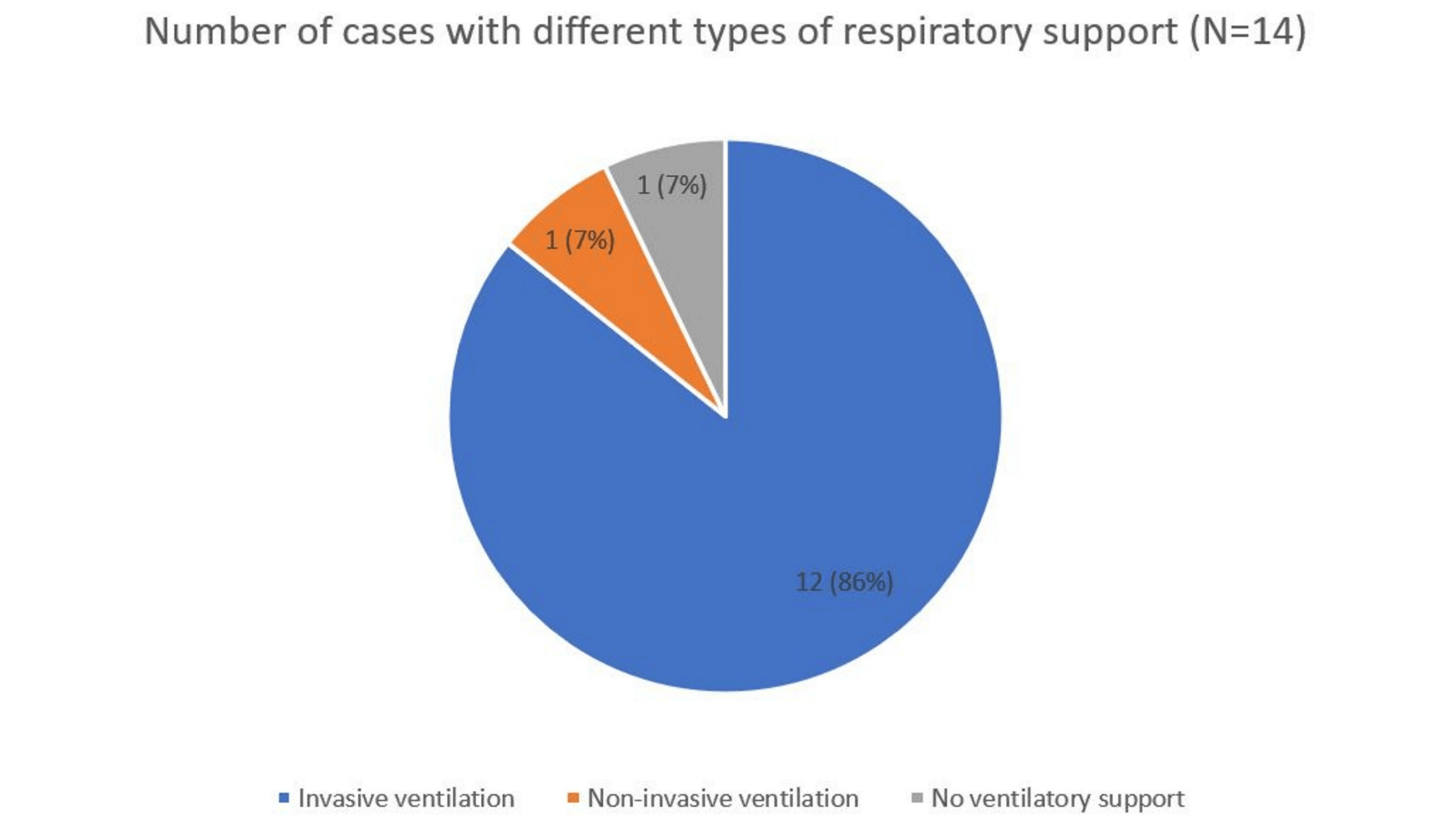
Number of cases with different types of respiratory support (N=14) Figure created by the authors.

Of the 14 reviewed cases (N = 14), 12 patients (86%) required endotracheal intubation in addition to potassium replacement therapy. Notably, a 2021 case report described successful use of non-invasive ventilation (NIV) in an intensive care unit setting, suggesting a potential role for NIV in selected patients to avoid complications associated with invasive ventilation [30]. Figure 5 illustrates the variation in the need for intubation versus NIV among these cases. 

Overall, the prognosis for TPP with respiratory muscle involvement was favorable, with most patients achieving full recovery. However, two cases resulted in death [23,34].

Respiratory Failure in Other Forms of Hyperthyroidism

Although this review primarily focuses on TPP and respiratory failure, an association between respiratory failure and thyroid storm is also briefly discussed.

Patients presenting with thyroid storm often exhibit respiratory involvement. Inspiratory muscle fatigue and failure are common in thyroid storm, which can lead to respiratory acidosis and necessitate mechanical ventilation. In thyrotoxicosis, muscle mass is decreased by approximately 20%, and muscle strength is reduced by about 40%. Thyrotoxic myopathy is thought to result from damage to the motor end plates. Additionally, active Graves’ disease is associated with functional weakness of the diaphragm. As respiratory muscles weaken, greater energy is required to maintain effective breathing, leading to fatigue. Ultimately, muscle fatigue results in respiratory failure characterized by carbon dioxide retention and acute hypercapnic respiratory failure [36].

Several mechanisms can contribute to acute respiratory failure in thyroid storm, including acute pulmonary edema, pulmonary embolism, thyrotoxic myopathy, thyrotoxic periodic paralysis, rhabdomyolysis, polymyositis, or coexisting myasthenia gravis, all of which may necessitate mechanical ventilation [37].

Thyroid storm almost always occurs in the setting of an underlying hyperthyroid state, whether overt or subclinical. In patients with pre-existing hyperthyroidism, systemic stressors such as infection, trauma, surgery, pregnancy, myocardial infarction, or certain medications can precipitate a thyroid storm. Without prompt treatment, thyroid storm can result in fatal cardiac and respiratory decompensation [38].

Discussion

The mechanisms underlying respiratory failure differ between TPP and thyroid storm. In TPP, respiratory muscle weakness is primarily related to hypokalemia-induced paralysis. In contrast, respiratory failure in thyroid storm predominantly results from muscle fatigue caused by thyrotoxic myopathy. Moreover, thyroid storm is characterized by multisystem involvement, including cardiopulmonary dysfunction, and is frequently precipitated by factors such as infection, stress, or trauma. Notably, TPP may also occur during thyroid storm due to the underlying hyperthyroid state.

Most reported TPP cases initially present with acute limb weakness, followed by respiratory muscle involvement. Early diagnosis of TPP can be challenging because initial symptoms often mimic neurological disorders, potentially leading to misdiagnosis. Similarly, in thyroid storm, precipitating medical conditions may obscure its classic clinical features, delaying accurate diagnosis and appropriate treatment initiation. Consequently, clinicians might focus on treating the presenting illness without recognizing thyroid storm as the underlying cause.

This review highlights that respiratory failure in TPP is commonly associated with hypokalemia. However, some cases report respiratory failure despite normal serum potassium levels, revealing a significant clinical gap that warrants further investigation into the underlying mechanisms.

Atypical clinical features, such as psychosis and convulsions, underscore the importance of maintaining a high index of suspicion for TPP and hyperthyroidism in patients presenting with respiratory distress, even in the absence of classical hyperthyroid signs.

In cases of TPP with respiratory failure, correction of hypokalemia remains a cornerstone of management, alongside beta-blockers and antithyroid medications. In thyroid storm, multiorgan dysfunction - including respiratory failure, high-output heart failure, and hepatic impairment - is common. Treatment typically involves antithyroid agents, corticosteroids, and beta-blockers, with plasma exchange considered in severe or refractory cases.

Invasive mechanical ventilation remains the mainstay for managing acute respiratory failure in TPP. However, where resources permit, NIV has shown promise when applied to hemodynamically stable patients in conjunction with potassium correction, potentially reducing complications associated with invasive ventilation [30]. Further research is needed to establish optimal timing and duration of NIV use in TPP.

Given the rarity but potential severity of TPP-associated respiratory failure, early recognition, prompt intervention, and multidisciplinary management involving respiratory, endocrinology, and intensive care specialists are critical to achieving favorable patient outcomes.

## Conclusions

Thyrotoxic periodic paralysis with respiratory failure is a rare but potentially life-threatening complication, most often resulting from hypokalemia-induced muscle weakness. Early recognition and prompt correction of electrolyte disturbances, combined with appropriate respiratory support, are critical for improving patient outcomes. While invasive mechanical ventilation is frequently required, emerging evidence suggests that early and carefully selected use of non-invasive ventilation may be beneficial in stable patients, potentially avoiding the risks of intubation. Moreover, respiratory failure in a hyperthyroid state is not limited to TPP. Thyroid storm, a multisystem emergency, can independently precipitate respiratory failure through various mechanisms such as myopathy, diaphragmatic muscle fatigue and cardiopulmonary complications. The complexity and overlapping mechanisms between these two conditions highlight the need for high suspicion in patients presenting with acute unexplained weakness and respiratory distress. Further research is warranted to elucidate the mechanisms underlying respiratory failure in cases with normal serum potassium levels and to establish clear criteria for the initiation of non-invasive ventilation in the management of TPP-associated respiratory failure. Multidisciplinary involvement - including endocrinology, respiratory medicine, and intensive care - is essential to optimize outcomes in this challenging but treatable condition.
